# *Escherichia coli* YcaQ is a DNA glycosylase that unhooks DNA interstrand crosslinks

**DOI:** 10.1093/nar/gkaa346

**Published:** 2020-05-15

**Authors:** Noah P Bradley, Lauren A Washburn, Plamen P Christov, Coran M H Watanabe, Brandt F Eichman

**Affiliations:** Department of Biological Sciences, Vanderbilt University, Nashville, TN 37232, USA; Department of Chemistry, Texas A&M University, College Station, TX 77843, USA; Institute of Chemical Biology, Vanderbilt University, Nashville, TN 37232, USA; Department of Chemistry, Texas A&M University, College Station, TX 77843, USA; Department of Biological Sciences, Vanderbilt University, Nashville, TN 37232, USA; Department of Biochemistry, Vanderbilt University, Nashville, TN 37232, USA

## Abstract

Interstrand DNA crosslinks (ICLs) are a toxic form of DNA damage that block DNA replication and transcription by tethering the opposing strands of DNA. ICL repair requires unhooking of the tethered strands by either nuclease incision of the DNA backbone or glycosylase cleavage of the crosslinked nucleotide. In bacteria, glycosylase-mediated ICL unhooking was described in *Streptomyces* as a means of self-resistance to the genotoxic natural product azinomycin B. The mechanistic details and general utility of glycosylase-mediated ICL repair in other bacteria are unknown. Here, we identify the uncharacterized *Escherichia coli* protein YcaQ as an ICL repair glycosylase that protects cells against the toxicity of crosslinking agents. YcaQ unhooks both sides of symmetric and asymmetric ICLs *in vitro*, and loss or overexpression of *ycaQ* sensitizes *E. coli* to the nitrogen mustard mechlorethamine. Comparison of YcaQ and UvrA-mediated ICL resistance mechanisms establishes base excision as an alternate ICL repair pathway in bacteria.

## INTRODUCTION

DNA damage arising from a variety of endogenous and environmental agents pose a significant risk to cell viability. Interstrand DNA crosslinks (ICLs) are highly toxic DNA lesions that covalently tether the opposing strands of DNA, thereby inhibiting essential cellular processes such as DNA replication and transcription that rely on duplex unwinding (1). ICLs are produced by highly abundant endogenous metabolites and DNA repair intermediates, and by a number of environmental toxins including microbial and plant natural products (e.g. mitomycin C and psoralens) that have therapeutic properties (2–6). Because of their cytotoxicity, crosslinking agents are often potent antimicrobials and many, including nitrogen mustards, are among the most widely used drugs in cancer chemotherapy (7,8).

ICL repair involves unhooking the two strands by one of two known pathways, followed by repair of the resulting monoadduct (3,9). The primary mechanism of ICL unhooking involves incisions to one strand by Fanconi anemia (FA) or nucleotide excision repair (NER) associated endonucleases (10–12). The resulting gap and strand break must be further processed by translesion DNA synthesis and homologous recombination prior to repair of the monoadduct by a second round of NER. An alternative ICL repair pathway was recently discovered in both eukaryotes and prokaryotes, whereby the ICL is unhooked by DNA glycosylase cleavage of one of the *N*-glycosidic bonds linking the modified nucleotide to the DNA backbone, generating an abasic (AP) site on one strand but leaving the backbone intact (13–15). DNA glycosylases typically initiate base excision repair (BER) of small monoadducts. Because their mode of lesion recognition and excision involves extruding (or flipping) a single nucleotide out of the helix, the mechanism by which they unhook ICLs tethered across strands is not understood.

Glycosylase-mediated ICL repair in bacteria was discovered in the biosynthetic gene cluster of azinomycin B (AZB), a genotoxic non-ribosomal peptide/polyketide secondary metabolite produced by the soil-dwelling microbes *Streptomyces sahachiroi* and *S. griseofuscus* (16). Azinomycins are bifunctional DNA alkylating agents that form ICLs between *N*7 of purines in 5′-PuNPy-3′ sequences (17). AZB displays potent antibacterial activity against a variety of bacterial species (7), antitumor activity at lower doses than mitomycin C (18), and initiates transcription of DNA damage response genes after treatment (19). The AZB synthesis cluster contains a novel DNA glycosylase, AlkZ, which cleaves AZB-ICLs and *N*7-methylguanine monoadducts *in vitro* to produce an AP site that can be processed by the bacterial AP endonuclease EndoIV (14,20). *AlkZ* expression is induced during production of AZB, and cells that express *alkZ* are resistant to the cytotoxic effects of AZB, even across different bacterial species (14). Thus, the AlkZ glycosylase provides self-resistance to the toxicity of these compounds in the producing organism (14,15).

AlkZ belongs to the functionally uncharacterized HTH_42 superfamily of bacterial proteins known only for their arrangement of tandem winged helix-turn-helix (wHTH) motifs commonly found in transcription factors and other DNA binding proteins (21). The crystal structure of AlkZ revealed a novel fold in which three wHTH motifs scaffold a C-shaped protein with a positively charged, concave surface that contains several residues important for DNA glycosylase activity (20) (Supplementary Figure S1). HTH_42 family proteins are widespread among antibiotic producers and human pathogens, including *Escherichia coli*, *Clostridiumdifficile*, and *Staphylococcus aureus*, among others (14). Whether these proteins are bona fide AlkZ homologs that contain ICL unhooking activity is not known.

To better understand the mechanism of glycosylase-mediated ICL repair and to determine whether glycosylase ICL repair in bacteria is limited to AZB production and resistance, we compared ICL unhooking activity of *Streptomyces* AlkZ and the uncharacterized *E. coli* homolog YcaQ for different types of ICLs. Our results indicate that YcaQ is a cationic alkylpurine DNA glycosylase with robust activity for a broad range of substrates, including nitrogen mustard ICLs, whereas *Streptomyces* AlkZ is specific for AZB-ICLs. Deletion of *ycaQ* from *E. coli* increased cellular sensitivity to the crosslinking agent mechlorethamine, and this phenotype was complemented by recombinant YcaQ. Furthermore, overexpression of *ycaQ* showed a strong sensitivity to both ICL and methylating agents that was dependent on generation of toxic intermediates in the BER pathway. Taken together, this work identifies *E. coli* YcaQ as an ICL repair glycosylase and implicates YcaQ-mediated BER as an alternative ICL repair pathway in *E. coli*.

## MATERIALS AND METHODS

### Reagents

*Escherichia coli* uracil DNA glycosylase (UDG), formamidopyrimidine DNA glycosylase (Fpg), and endonuclease (Endo) IV were purchased from New England BioLabs. *Escherichia coli* AlkA was generously provided by Pat O’Brien (University of Michigan). Human AAGΔ83, *Schizosaccharomyces pombe* Mag1, and *Bacillus cereus* AlkD were purified as previously described (22–24). Expression vectors pBG103 and pHD116 were from the Vanderbilt University Center for Structural Biology, and pSF-OBX11 was from Millipore Sigma. DNA oligonucleotides (Supplementary Table S1) were purchased from Integrated DNA Technologies. *Escherichia coli* K-12 wild-type and knockout strains were purchased from the Keio *E. coli* knockout collection (Dharmacon, GE Healthcare). Unless otherwise noted, all chemicals were purchased from Sigma-Aldrich.

### AlkZ and YcaQ purification

The *ycaQ* gene was amplified by PCR from genomic DNA isolated from *E. coli* K-12 MG1655 cells and cloned into pBG103. His_6_-SUMO-YcaQ was overexpressed in *E. coli* Tuner (DE3) cells at 16°C for 18 hr in LB medium supplemented with 30 μg/ml kanamycin and 50 μM isopropyl β-d-1-thiogalactopyranoside (IPTG). Cells were lysed with sonication and cell debris removed by centrifugation at 45,000 × g at 4°C for 30 min. Clarified lysate was passed over Ni-NTA agarose equilibrated in buffer A [50 mM Tris•HCl pH 8.0, 500 mM NaCl, 20 mM imidazole and 10% (vol/vol) glycerol] and protein eluted in 250 mM imidazole/buffer A. Protein fractions were pooled and supplemented with 0.1 mM EDTA and 1 mM tris(2-carboxyethyl)phosphine (TCEP) before incubation with ∼0.5 mg of Rhinovirus 3C protease (PreScission) at 4°C overnight. Cleaved protein was diluted 10-fold in buffer B [50 mM Tris•HCl pH 8.0, 10% (vol/vol) glycerol, 1 mM TCEP, and 0.1 mM EDTA] and purified by heparin sepharose using a 0–1 M NaCl/buffer B linear gradient. Fractions were pooled and repassed over Ni-NTA agarose in buffer A, concentrated and filtered, and passed over Superdex 200 size exclusion resin equilibrated in buffer C [20 mM Tris•HCl pH 8.0, 100 mM NaCl, 5% (vol/vol) glycerol, 1 mM TCEP, and 0.1 mM EDTA]. Protein was concentrated to 150 μM (AlkZ) or 10 μM (YcaQ), flash-frozen in liquid nitrogen, and stored at −80°C. AlkZ and YcaQ mutants were generated using the Q5 Mutagenesis Kit (New England BioLabs). Mutant proteins were overexpressed and purified the same as wild-type.

### Synthesis of NM_8_

2,2′-(Ethane-1,2-diylbis(methylazanediyl))bis(ethan-1-ol) (**1**) was synthesized as follows. In a dry round bottom flask were placed 2-(methylamino)ethan-1-ol (24 g, 0.32 mol), 1,2-dibromoethane 30 g, 0.19 mol), potassium carbonate (44 g, 0.32 mol), and dry ethanol (200 ml). The reaction mixture was refluxed overnight, cooled, and half of the solvent was removed under vacuum in a rotary evaporator. The solid was filtered off and washed with dry ethanol. The solvents were removed under vacuum and the product distilled off at high vacuum to yield the product as a viscous liquid (4.6 g, bp 80–90°C at 15 mm Hg, 8%). Low resolution mass spectra (MS) were obtained on an Agilent series 1200 single quad ChemStation autosampler System using electrospray ionization (ESI) in positive mode. HPLC was performed on an Accucore C18 column (2.6 μm, 2.1 mm × 30 mm column) at 40°C, using a 40–90% acetonitrile/water with 0.1% trifluoracetic acid gradient for 1.5 min and a flow rate of 1.5 ml/min. ^1^H-NMR (d_4_-MeOH; 400 MHz) δ 3.69 (tr, 4H, *J* = 4 Hz), 2.51 (tr, *J* = 4 Hz, 4H), 2.41 (s, 4H), 2.29 (s, 6H); MS (ESI): mass calcd. for C_8_H_21_N_2_O_2_, 177.2.; *m*/*z* found, 177.3 [M+H]+, Rf = 0.18.

**Scheme 1.**
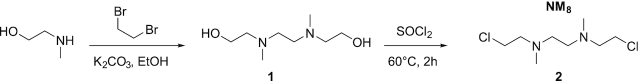
Synthesis of NM_8_.

The NM_8_ compound, *N*^1^,*N*^2^-bis(2-chloroethyl)-*N*1,*N*2-dimethylethane-1,2-diamine (**2**), was synthesized as follows. A dry round bottom flask containing thionyl chloride was cooled to −20°C, and 2,2′-(ethane-1,2-diylbis(methylazanediyl))bis(ethan-1-ol) (4.6 gr, 0.026 mol) was added dropwise with vigorous stirring. The mixture was heated at 60°C for 2 hr, excess thionyl chloride was removed in vacuum by rotary evaporator, and the solid was dried at high vacuum for 30 min. The solid was recrystallized from acetone/methanol to give pure product as hydrochloride salt (1.3 g, 20%). ^1^H-NMR (d_4_-MeOH) δ 4.00 (tr, 4H, *J* = 8Hz), 3.60 (m, 8H), 2.96 (s, 6H); ^13^C-NMR (d_4_-MeOH) δ 57.3, 49.9, 39.8, 36.7 (Scheme 1).

### Preparation of DNA substrates

DNA substrates containing a single d7mG lesion and a 5′-6-carboxyfluorescein (FAM) label within the sequence FAM-d(CACCACTACACC(7mG)ATTCCTTACAAC)/d(GTTGTAAGGAATCGGTGTAGTGGTG) were prepared as described previously (25). The mFaPy derivatives of 7mG-DNA and NM_5_-ICL-DNA were generated by treating 500 nM DNA with 100 mM NaOH at 37°C for 30 min, followed by re-adjusting to pH 7 with 100 mM HCl. The substrate to test excision of d7mG in proximity to an AP site was prepared by incubating unlabeled DNA with a sequence [d(GTTGTAAGGA**U**TCGGTGTAGTGGTC)] complementary to the FAM-labeled d7mG oligo with 1 U of UDG at 37°C for 30 min (Supplementary Figure S5B). AP-DNA was phenol-chloroform extracted and annealed to 12-fold molar excess of the complementary d7mG-DNA.

Azinomycin B was obtained using the fermentation protocol established previously (26). Briefly, *Streptomyces sahachiroi* cultures were centrifuged at 7000 rpm at 4°C, cell pellets discarded, and the medium extracted with an equal volume of dichloromethane. The organic layer was collected, dried over anhydrous magnesium sulfate, and concentrated *in vacuo*. The resulting crude extract was stored at −80°C and supplemented with 10% methanol prior to use. The presence of the AZB moiety was verified by HPLC-MS. AZB-ICLs were generated by annealing DNA with the sequence FAM-d(AAAAATAAAAGCCAAATAAAAATAAA) to the complementary oligo containing a 5′-Cyanine (Cy5) label. DNA (100 μM duplex) was incubated with 10 mg crude extract at 4°C on ice for 24 hr in the dark. The DNA was filtered through a 0.22 μm filter to remove debris from the extract and desalted using Illustra MicroSpin G25 columns (GE Healthcare) equilibrated in TE buffer (10 mM Tris•HCl pH 8.0, 1 mM EDTA). AZB-DNA was purified by denaturing PAGE. Briefly, DNA was denatured at 55°C for 2 min in 5 mM EDTA pH 8.0, 80% (wt/vol) formamide, 0.5 mg/ml orange G and electrophoresed on a 15% (wt/vol) acrylamide/8 M urea denaturing gel pre-run in 0.5× TBE buffer. FAM (488 nm excitation, 526 nm emission) and Cy5 (633 nm excitation, 670 nm emission) fluorescence were detected from the gel using a Typhoon Trio variable mode imager (GE Healthcare). The band with the slowest electrophoretic mobility that contained both the FAM and Cy5 fluorescence was cut out and placed in 3500 MWCO dialysis tubing (Thermo Fischer Scientific) with 2 ml of 0.5× TBE, and the DNA electroeluted from the gel at 120 V for 1 hr. The ICL-DNA were concentrated to 4 μM using an Amicon Ultra-3K filter (3500 rpm, 4°C, 45 min), buffer exchanged into TE buffer, aliquoted, and stored at −80°C.

NM-ICL DNA substrates were generated and purified similar to an established protocol (27). DNA with the sequence FAM-d(AAAAATAAAAGTCAAATAAAAATAAA) was annealed to the complementary Cy5-oligodeoxynucleotide at 100 μM duplex in 40 mM sodium cacodylate (pH 7.0). Crosslinks were generated by incubating the DNA with 300 μM mechlorethamine•HCl or NM_8_ compound (**2**) for 3 hr at 37°C while shaking in the dark. Unreacted drug was removed using a G25 spin column equilibrated in TE buffer, and NM-ICL-DNA gel purified the same as AZB-DNA.

### Base excision assays

In each glycosylase reaction, 1 μM enzyme was incubated with 100 nM FAM-DNA in glycosylase buffer [50 mM HEPES pH 8.5, 100 mM KCl, 10 mM EDTA, and 10% (vol/vol) glycerol] at 25°C. At various time points, 4-μl aliquots were added to 1 μl of either 1M NaOH and heated at 70°C for 2 min or 83 nM EndoIV and incubated at 37°C for 5 min. To test for AP lyase activity, the 4-μl aliquots were added to 1 μl glycosylase buffer and incubated at 70°C for 2 min. Samples were then denatured at 70°C for 5 min in 5 mM EDTA pH 8.0, 80% (wt/vol) formamide, 1 mg/ml blue dextran, and electrophoresed on a 20% (wt/vol) acrylamide/8 M urea sequencing gel at 40 W for 1 hr in 0.5× TBE buffer (45 mM Tris, 45 mM borate, and 1 mM EDTA pH 8.0). Gels were imaged on a Typhoon Trio variable mode imager (GE Healthcare) using 488-nm excitation and 526-nm emission fluorescence, and bands were quantified with ImageQuant (GE Healthcare). All excision assays were performed in triplicate.

Base excision unhooking of AZB- and NM-ICLs used a modified version of the d7mG protocol. Positive controls for ICLs involved heating the lesions to 95°C for 5 min to fully depurinate the DNA, followed by work-up with either water or NaOH to nick the backbone. To perform β/δ-elimination of AP-DNA products, samples were incubated with either 0.2 M NaOH at 55°C for 2 min or 17 nM EndoIV at 37°C for 5 min. Samples were denatured at 55°C in loading buffer for 5 min prior to electrophoresis. Gels were imaged for both FAM and Cy5 fluorescence and quantified individually. ICL unhooking was quantified by summing the individual FAM and Cy5 fluorescence intensities. The raw gels were artificially colored using Adobe Photoshop and overlaid using ImageJ software. Native PAGE analysis of AZB-ICL and NM-ICL excision products were carried out by suspending reaction mixtures in 2× native loading buffer [30% (vol/vol) glycerol, 4 mg/ml blue dextran, 400 mM NaCl, 100 mM Tris•HCl (pH 8.0) and 10 mM EDTA] and running on an 15% (wt/vol) acrylamide sequencing gel at 5 W for 3 hr. Double-stranded DNA standards were annealed in SSC buffer (300 mM NaCl and 30 mM trisodium citrate pH 7.0).

### Cellular assays

Genetic knockouts of *ycaQ* and *uvrA* were obtained from the *E. coli* Keio knockout collection (Dharmacon, GE Healthcare) that contained a kanamycin resistance cassette in place of the endogenous gene. The kanamycin resistance cassettes were removed using a FLP recombinase expressed on a temperature-sensitive pCP20 plasmid (Amp^R^). *ΔuvrA*/*ΔycaQ E. coli* were generated by recombineering through knockout of *ycaQ* in λ-Red competent *ΔuvrA* cells (λ-Red carried on temperature-sensitive pKN208 plasmid- Kan^R^). To generate *E. coli* growth curves, overnight cultures were diluted to 0.01 OD_600_ in LB supplemented with either 0 μM or 33 μM mechlorethamine in a 96-well flat-bottom plate. The plate was incubated at 30°C with shaking for 11 hr and cell density was measured at 600 nm every 20 min using a Bio-Tek Synergy 2 microplate reader.

*E. coli* survival curves after MMS or mechlorethamine treatment were performed using a colony dilution assay. *YcaQ* was overexpressed from a modified pBG103 (Kan^R^) vector and *nfo* (EndoIV) was overexpressed from a modified pHD116 (Amp^R^) vector, and expression confirmed by SDS-PAGE of cell lysates after IPTG induction (Supplementary Figure S6B). A saturated overnight culture from a single colony was diluted to 0.01 OD_600_ in fresh LB media and grown to 0.4 OD_600_ at 37°C, IPTG added to 100 μM, and the cells incubated at 37°C for 1 hr. The cells were transferred to fresh media and treated with various concentrations of either MMS or mechlorethamine for 2 hr at 37°C. Treated cells were transferred to fresh media, serially diluted by a factor of 10^−5^ or 10^−6^ in LB media, and 100 μl of diluted cells were plated on LB agar plates and grown at 37°C overnight. Colonies were counted the next morning and the CFU/ml of culture was determined. The percent survival was calculated as CFU/ml_Treated_/CFU/ml_Untreated_. Curves were fit to single exponential and EC_50_ values determined by the half-time, *t*_1/2_. For the genetic complementation experiments, *ycaQ* was expressed from a pSF-OBX11 (Kan^R^) plasmid that allows for constitutive gene expression at intermediate levels, and LB media was supplemented with 30 μg/ml kanamycin to retain the plasmid.

### Detection of gene expression by quantitative RT-PCR

Saturated *E. coli* K-12 cultures from a single colony were diluted to 0.01 OD_600_ in LB media and grown at 37°C to an OD_600_ of 0.5, after which MMS (5 mM) or mechlorethamine (200 μM) was added and cultures grown at 37°C for an additional 2 hr. Cultures were lysed with TRIzol reagent and the RNA extracted with phenol-chloroform and precipitated with isopropanol/ethanol as in (28). Residual genomic DNA was removed from the RNA by treatment with DNase I (New England BioLabs). The RNA was re-extracted from the reaction mixture and quantified by absorbance at 260 and 280 nm. The specificity of the primers and quality of the RNA was verified by agarose gel analysis of RT-PCR products (Supplementary Figure S6F). cDNA synthesis and qPCR were performed in a single step reaction using the iTaq Universal SYBR Green One-Step Kit (Bio-Rad) on a BioRad CFX-96 real-time PCR thermal cycler. The housekeeping gene used was *gapA* (*Eco* GAPDH). The results from the qPCR experiments were performed on three biologically replicated RNA extractions from both MMS and mechlorethamine treatments. The fold expression change was calculated using the formula: (fold expression change) = 2^−ΔΔCt^, where Ct is the cycle threshold for amplification above baseline, ΔCt = Ct (gene of interest) – Ct (housekeeping gene), and ΔΔCt = ΔCt (treated sample) – ΔCt (untreated sample).

## RESULTS

### *E. coli* YcaQ is an alkylpurine DNA glycosylase

**Figure 1.**
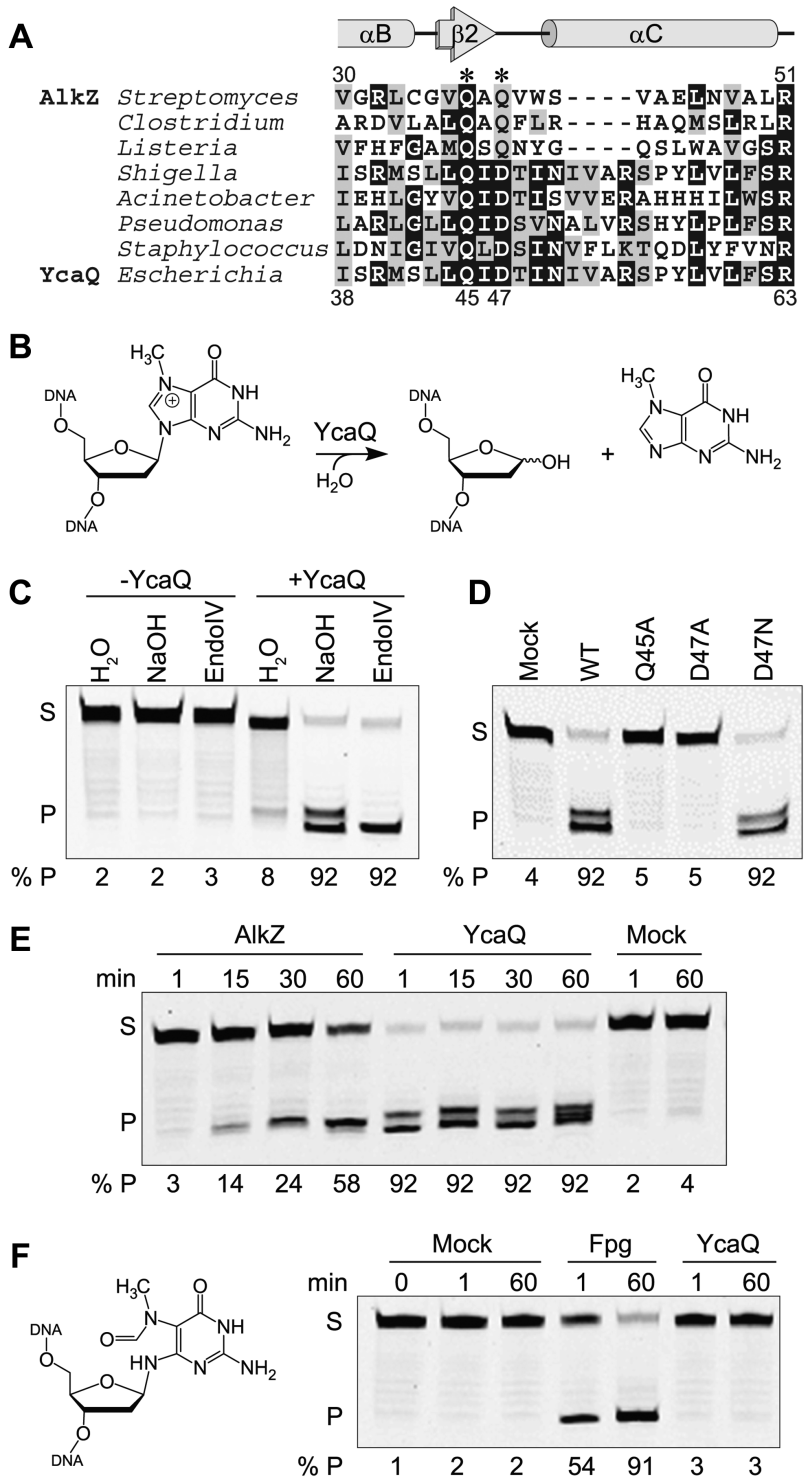
YcaQ is a Monofunctional DNA Glycosylase Specific for Cationic *N*-Alkylpurines. (**A**) Multiple sequence alignment of AlkZ/YcaQ homologs from *Streptomyces sahachiroi* and representative pathogens. Catalytic residues in AlkZ are labeled with asterisks. Secondary structures derived from the AlkZ crystal structure are shown above the alignment. (**B**) Schematic of d7mG base excision reaction. (C–F) Denaturing PAGE of 5′-FAM labeled d7mG-DNA substrate (S) and nicked AP-DNA product (P) after treatment with enzyme or buffer (mock). AP-DNA resulting from glycosylase activity was nicked by treatment with either 0.1 M NaOH (C–F) or EndoIV (C) to produce β- and β,δ-elimination products, which are quantified below each gel. (**C**) d7mG-DNA was incubated with (+) or without (-) YcaQ for 1 hr followed by treatment with either water, NaOH, or EndoIV. (**D**) Comparison of WT and mutant YcaQ activity after 1 hr. (**E**) Time-dependence of d7mG excision by AlkZ and YcaQ. The presence of a single product band in the AlkZ reaction is likely a result of EndoIV contamination in the work-up buffer, consistent with our previous analysis (20). (**F**) Structure and excision of mFaPy-dG DNA, treated with *E. coli* Fpg, YcaQ, or buffer for the specified time, followed by alkaline hydrolysis.

We first set out to determine whether other members of the HTH_42 family were bona fide AlkZ orthologs with DNA glycosylase activity. A search of the Pfam database (29) revealed 4,650 AlkZ orthologs, with 37% from pathogenic organisms and 21% annotated in antibiotic producing bacteria, consistent with a previous analysis (14). The sequences fell into one of two clades that differ in the catalytic motif. We previously showed that the glycosylase activity of *S. sahachiroi* AlkZ is dependent on two glutamine residues within a QΦQ motif, in which Φ is a hydrophobic residue. However, about 55% of the putative orthologs have an aspartate in the third position (Figure 1A, Supplementary Figure S1). We focused on one of these QΦD sequences—the uncharacterized *ycaQ* gene found in *E. coli*, which shares 54% sequence similarity and 31% identity to *Streptomyces* AlkZ.

We first tested whether YcaQ has DNA glycosylase activity for a simple alkyl-DNA monoadduct using the same *N*7-deoxymethylguanosine (d7mG) substrate tested previously against AlkZ (Figure 1B) (20). Purified YcaQ was incubated with d7mG-DNA containing a 5′-FAM label, followed by treatment with either hydroxide or the bacterial AP endonuclease, EndoIV, to nick any resulting AP sites. Compared to a no-enzyme control, YcaQ exhibited robust monofunctional base excision activity, as over 90% of the YcaQ treated substrate showed alkaline or AP endonuclease dependent cleavage (Figure 1C). We confirmed the importance of the putative QΦD catalytic motif by showing that alanine substitution of either Gln45 or Asp47 abrogated 7mG excision (Figure 1D). Mutation of Asp47 to asparagine did not affect base excision activity (Figure 1D), consistent with a functional carboxamide in this position in the QΦQ family. Compared to AlkZ, YcaQ displayed much faster 7mG excision kinetics, taking the reaction to completion in under one minute (Figure 1E) (20). We also tested wither YcaQ requires a cationic d7mG lesion for activity by converting the d7mG in our substrate to a ring-opened, neutral methylformamidopyrimidine (mFaPy) derivative (Figure 1F). Compared to a FaPy DNA glycosylase (Fpg) control, YcaQ had no excision activity for the mFaPy adduct, even after one hour (Figure 1F). Together these results show that YcaQ is a monofunctional DNA glycosylase from *E. coli* that can excise cationic d7mG monoadducts with rapid kinetics.

### AlkZ and YcaQ unhook either side of an AZB-ICL

**Figure 2.**
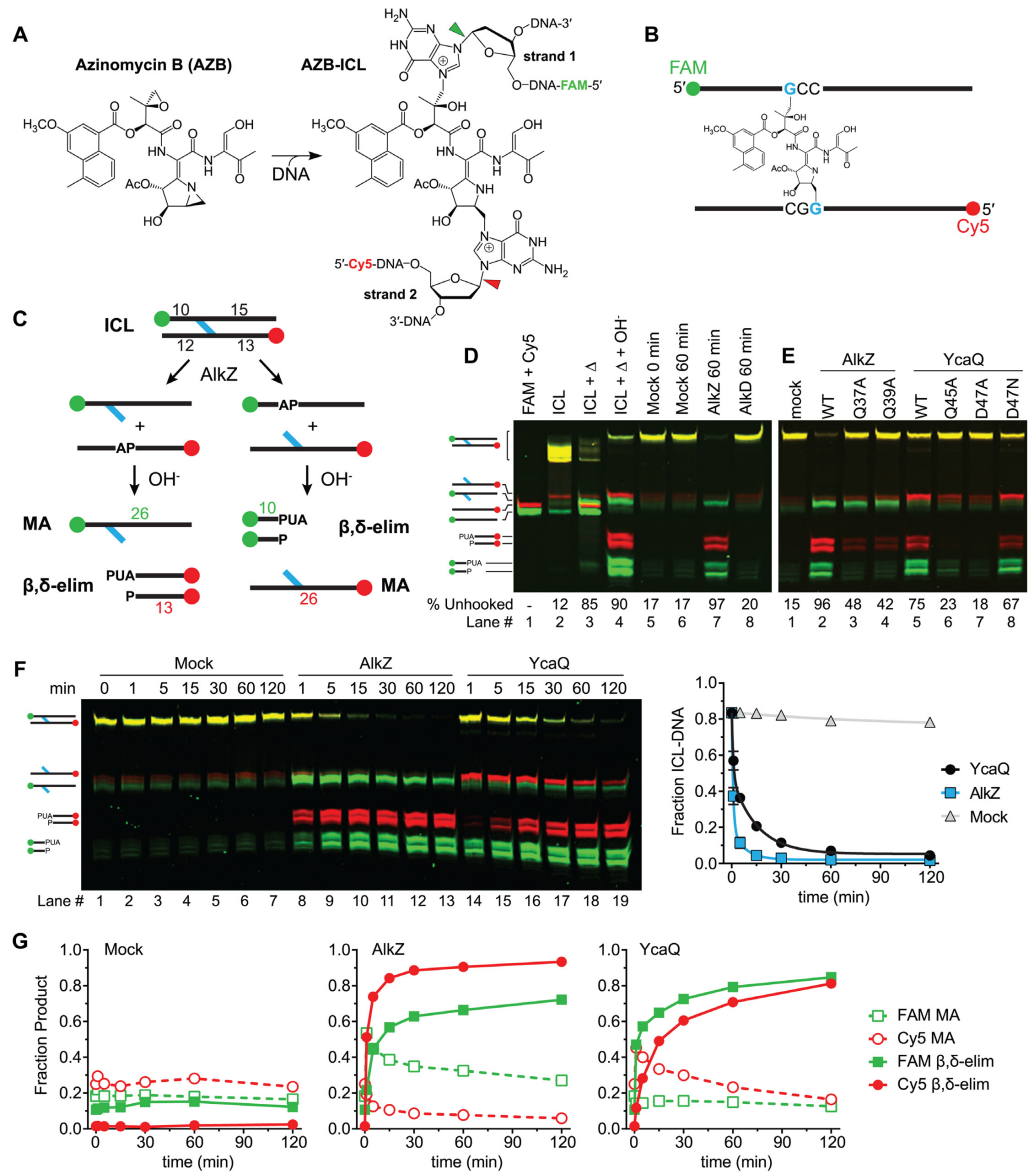
AlkZ and YcaQ can unhook either side of an azinomycin B-ICL. (**A**) Reaction of azinomycin B with guanines on opposing strands to form an ICL. *N*-glycosidic bonds hydrolysed by AlkZ/YcaQ are highlighted with green and red arrows, corresponding to FAM and Cy5-labeled strands, respectively. (**B**) Schematic of the 26-mer oligonucleotide AZB-ICL substrate used in this study. The epoxide predominantly reacts with the FAM-labeled GCC strand and aziridine with the Cy5-labeled GGC strand. Green and red spheres represent FAM and Cy5 labels, respectively. (**C**) Schematic of the base excision assay used to monitor ICL unhooking activity. Glycosylase unhooking of ICL-DNA potentially forms monoadducts and AP sites on either strand. AP-sites are nicked with hydroxide to form shorter oligonucleotides through β,δ-elimination. ICL-, monoadduct (MA)-, and β,δ-elimination products can be separated by denaturing PAGE. PUA, 3′-phosphor-α,β-unsaturated aldehyde (β-elimination product), P, 3′-phosphate (β,δ-elimination product). (**D-E**) Denaturing PAGE of AZB-ICL, AZB-MA, and nicked AP-DNA products after treatment with heat (Δ), buffer (mock), or enzyme, followed by alkaline hydrolysis. The percent of β,δ-elimination product is quantified below the gel. Each image is an overlay of false-colored FAM (green) and Cy5 (red) fluorescence scans of the gels, in which yellow depicts coincident red and green intensity. Individual FAM and Cy5 imaged gels are shown in Supplementary Figure S2. (**E**) 30-min reactions between AlkZ, YcaQ and catalytic mutants for AZB-ICL-DNA. (**F**) Denaturing PAGE showing time-dependence of AlkZ and YcaQ unhooking of AZB-ICLs. The fraction of ICL-DNA unhooked from three independent experiments is quantified to the right (mean ± SEM). (**G**) Quantification of the fraction of monoadduct (MA) and β,δ-elimination (nicked product) from the mock (left), AlkZ (middle), and YcaQ (right) reactions from the gel in panel F.

To examine ICL unhooking by the AlkZ/YcaQ family of glycosylases, we first compared their activities for AZB-ICLs. The azinomycins contain electrophilic epoxide and aziridine moieties that react with d(GCC)/d(GGC) sequences in a specific orientation to form an asymmetric ICL (Figure 2A) (30–32). AZB preferentially orients itself such that the epoxide moiety reacts with the GCC sequence and the aziridine reacts with GGC (Figure 2B). The previous study showing AZB-ICL cleavage by AlkZ did not address if the enzyme has a preference for unhooking one side versus the other (14). To better understand the mechanism of ICL unhooking, we devised a DNA substrate that would allow us to track individual strands. Pure AZB-ICL substrates for glycosylase assays were generated by incubating *S. sahachiroi* cell extracts with a 26-mer oligodeoxynucleotide duplex containing a single AZB reactive sequence, followed by denaturing PAGE purification to isolate the resulting crosslinked DNA. GCC and GGC strands were distinguished with FAM and Cy5 fluorescent labels at their 5′-ends, respectively, so that unhooking produces either FAM-AP + Cy5-monoadduct (MA) strands or FAM-MA + Cy5-AP strands (Figure 2C). In addition, the reactive sequence is offset toward one end of the duplex so that alkaline cleavage of the resulting AP sites will generate specific FAM- or Cy5-labeled β- and β+δ-elimination products that will migrate differently on a denaturing gel (Figure 2C).

Heat treatment of the purified AZB-ICL substrate at 95°C for 5 min resulted in cleavage of both sides of the crosslink, as evidenced by both FAM- and Cy5-labeled AP and MA strands that could be resolved on the gel (Figure 2D, lane 3). Treatment of the heat denatured ICLs with hydroxide converted the faster migrating AP strands into nicked β/δ-elimination products and left the MA strands intact (Figure 2D, lane 4). Alkali treatment also converted the ICL into a slower-migrating species, consistent with conversion of the double-cationic species to a neutral FaPy-ICL. Thus, AZB-ICLs display the same heat-labile depurination as other *N*7-guanine lesions (33). At 25°C, the AZB-ICL substrate is stable, showing no degradation after one hour (Figure 2D, lanes 5–6). Incubation with AlkZ under the same condition resulted in complete (97%) ICL unhooking into elimination products from both strands, indicating that AlkZ unhooks the AZB-ICL from either side. The persistence of a modest amount of FAM-labeled alkali-resistant MA strand suggested an enzymatic preference for unhooking the GGC side of the crosslink (Figure 2D, lane 7). Because the bases tethered by an ICL are constrained across the duplex, unhooking likely does not involve base-flipping as observed in other glycosylases. However, we found that the non-base flipping glycosylase AlkD (34,35) had no activity for the AZB-ICL substrate, indicating that ICL unhooking is not simply a product of a non-base-flipping mechanism and that recognition of AZB requires a specific type of enzyme.

YcaQ also unhooked the AZB-ICL substrate to generate AP sites on both strands (Figures 2E-G), albeit in a slightly different manner than AlkZ. Interestingly, whereas the FAM-MA strand persisted in the AlkZ reaction, YcaQ generated more Cy5-MA strand (compare WT lanes in Figure 2E), suggesting a difference in strand preference of the two enzymes. Second, alanine substitution of either residue in the catalytic QΦQ and QΦD motifs had different effects on unhooking. Mutation of QΦQ in AlkZ only partially reduced unhooking, while substitutions within YcaQ QΦD had a greater effect (Figure 2E), suggesting that the two enzymes rely on these motifs for catalysis to a different extent. Incidentally, the YcaQ D47N mutation had no effect on ICL unhooking activity, similar to its behavior against the d7mG monoadduct.

To further probe the apparent strand preference between AlkZ and YcaQ, we monitored the kinetics of AP and MA strand product formation over two hours under single-turnover conditions (Figure 2F,G). AlkZ showed robust AZB-ICL unhooking activity (*k*_cat_ = 2 × 10^−2^ s^−1^)—three orders of magnitude faster than that observed previously for a d7mG monoadduct (*k*_cat_ = 8.5 × 10^−5^ s^−1^) (20). YcaQ displayed 4-fold slower AZB-ICL unhooking kinetics under the same conditions (*k*_cat_ = 4 × 10^−3^ s^−1^) (Figure 2F). Quantification of products from both FAM and Cy5 strands shows that AlkZ has a distinct preference for unhooking the GGC (Cy5) strand, as shown by the greater burst in cleaved Cy5-AP and uncleaved FAM-MA strands (Figure 2F,G). In contrast, YcaQ had a modest preference for unhooking the GCC (FAM) strand (Figure 2F,G), but the differences in FAM and Cy5 strand kinetics in the YcaQ reaction were not as dramatic as in AlkZ. These data show that the orthologous YcaQ and AlkZ enzymes unhook AZB-ICLs from either side, and with unique strand specificities.

### YcaQ unhooks ICLs derived from nitrogen mustards

**Figure 3.**
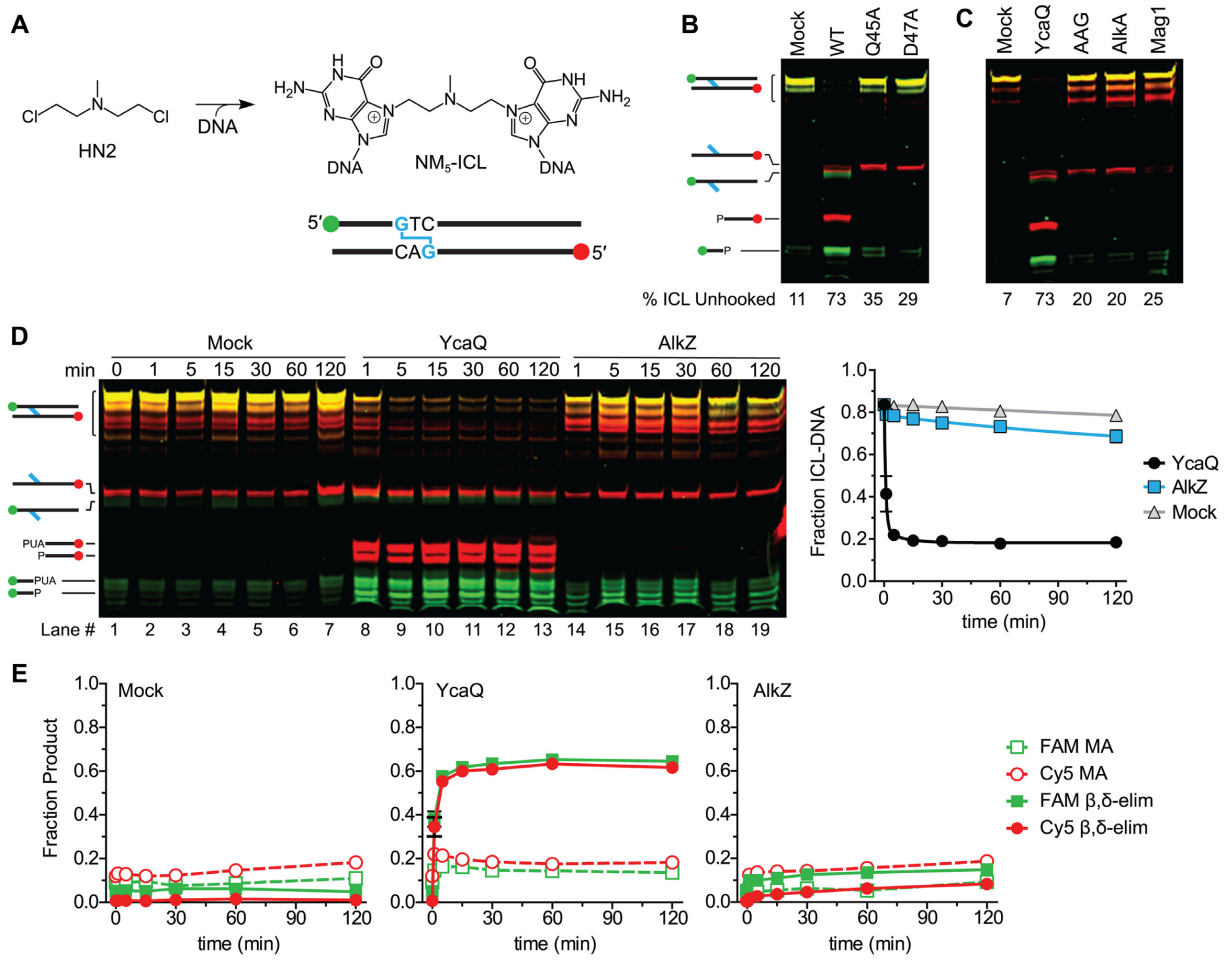
YcaQ unhooks nitrogen mustard ICLs. (**A**) 5-atom nitrogen mustard (NM_5_) ICL formed between mechlorethamine and guanines located on opposite DNA strands. (**B-C**) Denaturing PAGE of NM_5_-ICL substrate, monoadduct, and nicked AP-DNA products after treatment with WT or mutant YcaQ (B), or with YcaQ, human AAGΔ83, *E. coli* AlkA, or *S. pombe* Mag1 (C), followed by treatment with EndoIV. The percent of unhooked ICL is quantified below the gel. Images are false colored overlays of individual FAM and Cy5 scans of the gel, which are shown in Supplementary Figure S3. (**D**) Denaturing PAGE showing time-dependence of AlkZ and YcaQ unhooking of NM_5_-ICLs with a hydroxide workup. The plot quantifies the decrease in the fraction of NM_5_-ICL (mean ± SEM, *n* = 3). (**E**) Quantification of the fraction of monoadduct (MA) and β,δ-elimination (nicked AP-DNA product) from the mock (left), AlkZ (right), and YcaQ (middle) reactions from the gel in panel D.

AlkZ presumably evolved to provide self-resistance to AZB produced in soil-dwelling *S. sahachiroi*, whereas YcaQ in *E. coli* residing in the human gut would not encounter such a lesion. We therefore examined the ability of these enzymes to recognize an ICL derived from the nitrogen mustard (NM) mechlorethamine, a structurally simpler bifunctional alkylating agent than AZB (2). Like AZB, mechlorethamine reacts with *N*7 of both guanines within d(GNC) sequences to form ICLs that lead to the toxicity of these compounds (36) (Figure 3A). Using a site-specific NM-ICL oligonucleotide substrate (27) containing individually labeled strands (Figure 3A), we found that YcaQ unhooked either side (Figure 3B). We verified that this activity is specific to YcaQ, as β/δ-elimination products were not observed from QΦD (Q45A, D47A) catalytic mutants (Figure 3B), or from other alkylpurine DNA glycosylases, including human AAG, *E. coli* AlkA, and *Schizosaccharomyces pombe* Mag1 (Figure 3C; Supplementary Figure S3B). Furthermore, YcaQ showed no unhooking activity toward FaPy-ICLs (Supplementary Figure S3D,E), consistent with our results with mFaPy monoadducts indicating that the cationic, ring-closed guanine is required for the reaction. Most strikingly, comparison of the kinetics of NM-ICL unhooking showed that YcaQ was remarkably efficient (the reaction was complete in less than 5 minutes), whereas AlkZ showed no activity (Figure 3D). Unlike the asymmetric unhooking observed with the AZB-ICL, we did not detect a strand preference from the symmetric NM-ICL (Figure 3E). Comparison of the activities of both enzymes for AZB- and NM-ICLs shows that AlkZ is specific for AZB, whereas YcaQ can unhook chemically diverse ICLs, with robust activity toward the ICL with less chemical functionality.

**Figure 4.**
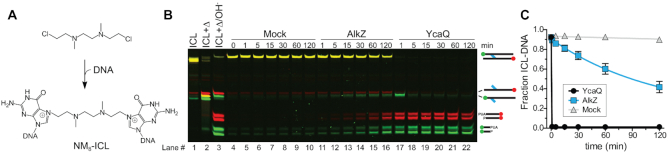
NM-ICL unhooking by YcaQ does not depend on a kinked duplex. (**A**) Structure and ICL formed from 8-atom nitrogen mustard (NM_8_) derivative. (**B**) Denaturing PAGE of the NM_8_-ICL-DNA substrate and nicked abasic-DNA products after treatment with buffer (mock), AlkZ or YcaQ for the specified time followed by alkaline hydrolysis. Heat-mediated depurination (lanes 2–3) serve as a positive control for excision products. Individual FAM and Cy5 imaged gels are shown in Supplementary Figure S4. (**C**) Quantification of the fraction of ICL from three separate experiments (mean ± SEM).

The mechanism of lesion recognition by a repair enzyme is a key question in ICL repair. Crosslinks may be located either by explicit interaction with the crosslinking molecule or by a structural perturbation of the DNA imposed by the crosslink. AZB, which has a 10-atom tether between guanines, is not expected to distort the DNA (31). In contrast, the 5-atom tether in the NM-ICL has been shown to kink the double helix (37,38). To determine whether the differences in ICL specificity of AlkZ and YcaQ are the result of DNA distortion, we synthesized a NM derivative that would produce an 8-atom ICL (NM_8_-ICL) when incorporated into our oligodeoxynucleotide substrate (Figure 4A), similar to that used previously (1). The increased tether length of the NM_8_-ICL should be able to crosslink the opposing guanines in B-form DNA without distorting the helix. Consistent with *N*7-alkylation at both sites, the purified NM_8_-ICL was heat-labile and fully depurinated from both strands after 5 min at 95°C (Figure 4B, lanes 1–3), but was stable at 25°C for at least 2 hr (Figure 4B, lanes 4–10). Compared to a no-enzyme control, AlkZ exhibited a low level of unhooking activity for the NM_8_-ICL substrate (Figure 4B,C, Supplementary Figure S4), suggesting that the steric strain present in the NM_5_-ICL at least partially inhibits AlkZ activity. However, AlkZ’s modest activity for the NM_8_-ICL was still much less than that of the AZB-ICL, and thus we conclude that explicit contact with the AZB moiety is an important aspect of ICL recognition by AlkZ. YcaQ showed strong activity toward the NM_8_-ICL as it did with the NM_5_-ICL (Figure 4B), indicating that a kinked duplex is not required for ICL unhooking by the *E. coli* enzyme.

### YcaQ and AlkZ create opposing AP sites *in vitro*

**Figure 5.**
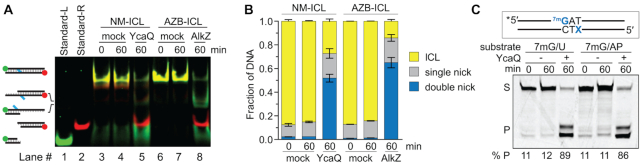
YcaQ and AlkZ create opposing AP sites. (**A**) Native PAGE analysis of DNA products formed from YcaQ incubation with NM_5_-ICL and AlkZ incubation with AZB-ICL DNA substrates. AP sites formed from glycosylase activity were nicked by incubation with EndoIV prior to loading the DNA. Double-stranded standards for the double-nicked excision products are shown in lanes 1–2. The gel is a false-colored composite of the individual FAM- and Cy5-imaged gel, which are shown in Supplementary Figure S5A. (**B**) Quantification of the total fluorescent signal for the fraction of ICL, single- and double-nicked EndoIV cleavage products from the gel in panel A. (**C**) Denaturing PAGE of d7mG/AP- and d7mG/dU-DNA substrates (S) and nicked AP-DNA products (P) after treatment with (+) or without (–) YcaQ followed by alkaline hydrolysis. Only the FAM-d7mG strand is visualized on the gel. The percent of β,δ-elimination products is quantified below.

Because AlkZ and YcaQ are able to act on either side of the crosslink, we asked whether they could act on both sides of the same crosslink. Such activity would generate two closely-spaced AP sites on opposite strands that in cells would potentially lead to a double-strand break (DSB). Indeed, the initial characterization of AlkZ activity toward AZB-DNA provided evidence that the enzyme produces opposing AP sites that can be subsequently cleaved by EndoIV (14). To further test this, we analyzed the products of EndoIV-treated AlkZ/AZB-ICL and YcaQ/NM-ICL reactions by native PAGE to quantify single- and double-nicked products. Both reactions showed at least 50% double-nicked product and a modest (10–20%) amount of monoadduct intermediate after one hour (Figure 5A, B, Supplementary Figure S5A), indicating that AlkZ and YcaQ cleave both sides of the crosslinks to generate AP sites on opposing strands. HPLC-MS analysis of the excision products of the YcaQ NM-ICL reaction confirmed the presence of an unmodified bis-guanine NM-ICL, with an observed m/z of 386.1799 Da, which is a 0.78 ppm difference from the calculated *m*/*z* of 386.1796 Da for Gua-NM_5_-Gua. The difference in rates of unhooking the two sides of the crosslink shown in Figure 2 argues that the strands are cleaved sequentially, which would proceed via an intermediate containing an AP site on one strand across from a monoadduct on the other. In support of this, we found that YcaQ is able to excise 7mG in a duplex containing an AP site on the opposite strand in the position it would reside in the unhooked NM- or AZB-ICL intermediate (Figure 5C).

### Deletion or overexpression of YcaQ sensitizes *E. coli* to crosslinking agents

**Figure 6.**
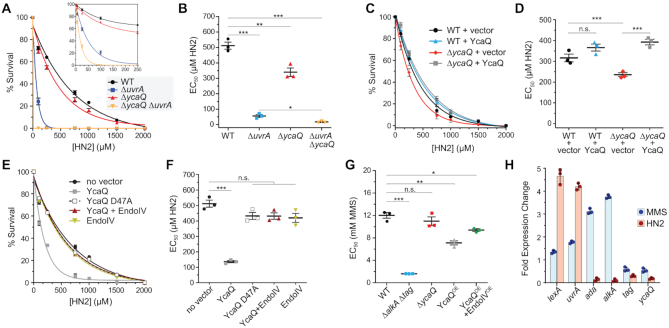
Deletion and overexpression of YcaQ sensitizes *E. coli* to mechlorethamine. (**A**) Colony dilution assay for *E. coli* deletion strains exposed to increasing concentrations of mechlorethamine (HN2). Values are mean ± SEM (*n* = 3). Percent (%) survival is relative to untreated cells. (**B**) EC_50_ values derived from data in panel A. Significance values were calculated using a one-way ANOVA (*P = 0.0140; **P = 0.0085; ***P < 0.0001; n.s, not significant). (**C**) Colony dilution assay for *E. coli* strains complimented with YcaQ or empty vector. Values are mean ± SEM (*n* = 3). (**D**) Quantification of the data shown in panel D. One-way ANOVA values: ***P = 0.0004, n.s., not significant. (**E**) Colony dilution assay showing the effect of mechlorethamine (HN2) on wild-type *E. coli* overexpressing YcaQ variants and/or EndoIV. (**F**) Quantification of the data shown in panel E. One-way ANOVA significance values: *P = 0.0088; **P = 0.0013; ***P < 0.0001; n.s, not significant. (**G**) MMS EC_50_ values (mM) for various *E. coli* deletion (Δ) or over-expression (OE) strains. (H) qRT-PCR results of DNA repair genes after treatment of *E. coli* with 5 mM MMS or 200 μM mechlorethamine (HN2) for 2 hr. Average ± SEM for three biological replicates.

Repair of mechlorethamine derived NM-ICLs in *E. coli* is known to be initiated by the UvrABC nucleotide excision system (39). Given the robust unhooking of NM-ICLs by YcaQ *in vitro*, we compared YcaQ’s role in protecting *E. coli* against mechlorethamine to that of UvrA using genetic knockouts. Relative to the wild-type strain, *ΔycaQ* cells showed a modest growth defect in the presence of low levels of mechlorethamine (Supplementary Figure S6A). As expected from the importance of UvrA in ICL repair, *ΔuvrA* cells showed a more severe growth deficiency. This sensitivity was exacerbated in a *ΔycaQΔuvrA* double knockout (Supplementary Figure S6A), suggesting that YcaQ plays a minor role in ICL repair in *E. coli*. To examine this more quantitatively, we determined EC_50_ values for the deletion strains exposed to increasing doses of mechlorethamine using a colony dilution assay (Figure 6A). Consistent with the growth sensitivity, *ΔycaQ* cells showed a modest but significant reduction in EC_50_ compared to wild-type, and sensitivity of the *ΔycaQΔuvrA* mutant was greater than for *ΔuvrA* alone (Figure 6B). Exogenous expression of *ycaQ* in the deletion strain rescued the mechlorethamine sensitivity compared to an empty vector control (Figure 6C, D). Thus, YcaQ provides a bona fide mechanism for cellular protection against ICL toxicity, albeit to a lesser extent than the UvrABC system.

We also found that overexpression of *ycaQ* exhibited a dramatic increase in mechlorethamine sensitivity, with an EC_50_ value approaching that of *ΔuvrA* cells (Figure 6E, F). This increased sensitivity was dependent on the catalytic activity of YcaQ, as overexpression of the inactive D47A mutant had no effect. Sensitivity to DNA damaging agents from DNA glycosylase overexpression is known to result from overwhelming the cells with AP sites and other toxic BER intermediates (40–43). Consistent with this interpretation, overexpression of both *ycaQ* and the AP endonuclease *endoIV* (*nfo*), which would help clear AP sites via BER, rescued the sensitivity (Figure 6E, F; Supplementary Figure S6B). These results further implicate BER as a mechanism of ICL repair in bacteria.

Because YcaQ also displays activity toward alkyl monoadducts, we tested the effect of *ycaQ* deletion and overexpression on cells challenged with the methylating agent methylmethanesulfonate (MMS). *E. coli* contains only two other alkyl DNA glycosylases, the constitutively active Tag and inducible AlkA (44,45). We found that the *ΔycaQ* strain was no more sensitive to MMS than wild-type cells, in stark contrast to the effect of knocking out both Tag and AlkA (Figure 6G; Supplementary Figure S6C, D) (46,47). Overexpression of *ycaQ* increased cellular sensitivity towards MMS, although not to the same extent as observed for mechlorethamine treatment (Figure 6G; Supplementary Figure S6E). This sensitivity was partially rescued by overexpression of both *ycaQ* and *endoIV*, again consistent with *ycaQ* overexpression leading to generation of toxic BER intermediates.

### YcaQ is constitutively expressed in cells

Prokaryotes have several inducible DNA damage responses to crosslinking and alkylating agents (48,49). To test if *ycaQ* expression is induced by ICL agents, quantitative RT-PCR was performed on *E. coli* cells exposed to mechlorethamine (Supplementary Figure S6F). As part of the SOS response to crosslinking agents, the NER *uvr* genes are under the control of the *lexA* repressor. Both *lexA* and *uvrA* showed a robust increase in gene expression upon mechlorethamine treatment, whereas *ycaQ* expression remained unchanged after treatment (Figure 6H). We also tested whether *ycaQ* is induced by the methylating agent MMS. The adaptive response genes, *ada* and *alkA*, were used as positive controls and the constitutively expressed glycosylase *tag* served as a negative control. Whereas both *ada* and *alkA* showed elevated mRNA levels, we found that *ycaQ* expression is not induced by MMS (Figure 6H). Together, these results indicate that *ycaQ* gene expression is not induced by either of the ICL or alkylating agents tested.

## DISCUSSION

YcaQ is the third alkylpurine DNA glycosylase to be identified in *E. coli* (50). Unlike Tag and AlkA, which excise only monoadducts during BER (51,52), YcaQ acts on crosslinked nucleobases to provide an alternative to NER-coupled ICL repair. The apparent specificity of YcaQ for cationic alkylpurines would limit the types of ICLs to be repaired to those formed by alkylation at purine *N*3- or *N*7 positions, such as nitrogen mustards. YcaQ showed a much greater activity toward unhooking NM-ICLs than other known alkylpurine DNA glycosylases. This is somewhat contrary to a previous report that AlkA and AAG act on mustard-treated DNA, although that study did not distinguish between ICL and monoadducts (53). Cells lacking *ycaQ* displayed a slight sensitivity towards mechlorethamine, but not MMS, consistent with the redundancy in repair mechanisms against methyl-DNA lesions (54). Repair of NM-ICLs in *E. coli* is known to depend on NER and HR pathways, as *uvrA* and *recA* mutants show extreme sensitivity towards crosslinking agents (55,56). The greater sensitivity of *ΔuvrA* versus *ΔycaQ* mutants to mechlorethamine shown here is consistent with NER/HR as the major ICL repair pathway for this type of lesion in *E. coli*.

A glycosylase-mediated ICL unhooking pathway could provide an error-free ICL repair mechanism by potentially bypassing the requirement for error-prone TLS across from the monoadduct (15). However, our *in vitro* data indicate that both AlkZ and YcaQ unhook crosslinks from either side to generate opposing AP sites in a 1,3 orientation that can be incised by EndoIV. In the cell, opposing AP sites in such close proximity could potentially lead to a deleterious DSB. Generation of opposing AP sites by AlkZ/YcaQ is in stark contrast to the 5mC glycosylase DEMETER in plants that removes 5mC in hemi-methylated d(5mCG/CG) islands and is inhibited by closely spaced 5mC residues on opposite strands in fully methylated d(5mCG)_2_ sequences (57). Our data suggest a sequential mechanism of unhooking both sides of the ICL. We do not know if the enzyme dissociates and rebinds after the first unhooking event or whether an enzyme-DNA complex persists to make the second cut, although structural modeling of the AlkZ/DNA complex shows a potential for crosstalk between two enzymes bound on either side of the crosslink (15,20). It remains to be determined in a cellular context if YcaQ forms opposing AP sites or DSBs, or if the enzyme is instead regulated to avoid a detrimental outcome. It also remains to be determined what the downstream resolution of glycosylase-mediated repair is, although previous studies suggest that *polB* (DNA pol II) may be involved in a recombination-independent mechanism for repair of NM-ICLs in *E. coli* (55,56).

In eukaryotes, the NEIL3 glycosylase was identified as an alternate ICL pathway to FA/NER and capable of unhooking ICLs derived from abasic sites and psoralen (13,58,59). NEIL3 and YcaQ are structurally unrelated and likely do not share the same mode of ICL recognition (20,60). ICL repair by the NEIL3 glycosylase is S-phase dependent and involves convergent replication forks (13,61), and its glycosylase domain has an intrinsic specificity for DNA damage at one particular orientation of forked structures (62). In contrast, the data presented here suggest that the bacterial AlkZ/YcaQ ICL glycosylase recognizes ICLs without regard to a specific DNA structure, consistent with ICL repair in bacteria occurring in the context of duplex DNA (3,63). Moreover, AlkZ’s preference for an AZB-ICL is likely the result of direct contact between the protein and AZB. Our AlkZ crystal structure modeled against AZB-DNA suggests that helix αI in the second wHTH motif is in proximity to make direct contact with the AZB moiety (Supplementary Figure S1) (20). This region differs in sequence between the *Streptomyces* and *E. coli* enzymes, consistent with its putative role in substrate specificity.

Our results indicate that *ycaQ* expression is likely constitutive as it was not induced by either of the ICL or alkylating agents tested. Consistent with constitutive expression, *ycaQ* resides within a putative four-gene operon containing essential genes *msbA* and *lpxK* behind a σ^70^-dependent promoter (Supplementary Figure S6G) (64). Large scale proteomic studies in *E. coli* suggest that YcaQ protein levels are among the lower 25% for abundance, indicating it may be kept at low protein concentrations in the cell (65). Interestingly, the *ycaQ* operon also has a putative σ^32^-dependent (heat shock) promotor, indicating a potential role for *ycaQ* and this operon in a global stress response (66). High-throughput transcriptomic studies of *E. coli* under different stresses shows *ycaQ* expression is upregulated during heat and cold shock (67). These data argue that YcaQ does not have a specific substrate, but instead is a mechanism to unhook diverse ICLs arising from various bifunctional alkylating agents. Nevertheless, we cannot rule out the possibility that YcaQ plays a specific role in certain strains of *E. coli* or other related bacteria that produce genotoxic secondary metabolites during a stress response. For example, *pks^+^ E. coli* producing colibactin, an ICL agent associated with formation of colon cancer (68–74), likely have some form of self-resistance against the toxin. YcaQ may play that role in a manner similar to that of *Streptomyces* AlkZ for azinomycin or of the AlkD/YtkR2 glycosylases for the non-covalent ICL agent yatakemycin (14,34,35). Although more work is needed to understand the rationale for YcaQ involvement in ICL repair in *E. coli* and other bacteria, this work expands the role of DNA glycosylases and the BER pathway in repair of ICLs.

## Supplementary Material

gkaa346_Supplemental_File

## Notes

Present address: Brandt F. Eichman, Department of Biological Sciences, Vanderbilt University, Nashville, TN 37235, USA.
