# Supplementary material for: Escherichia coli YcaQ is a DNA glycosylase that unhooks DNA interstrand crosslinks
Source: Nucleic Acids Res. 2020 May 15;48(13):7005–17. doi: 10.1093/nar/gkaa346 (PMC7367128; doi:10.1093/nar/gkaa346)
Supplement: gkaa346_Supplemental_File [file gkaa346_supplemental_file.pdf]

**Supplemental Information**

# *Escherichia coli* YcaQ is a DNA glycosylase that unhooks DNA interstrand crosslinks

Noah P. Bradley, Lauren A. Washburn, Plamen P. Christov, Coran M. H. Watanabe, Brandt F. Eichman*

**Table S1. Oligodeoxynucleotides used in this study**

| Oligo Name | Sequence (5′→3′)[a] | Use |
|---|---|---|
| YcaQ_FP_BamH1 | TCATCAGGATCCATGTCGCTGCCGCACCTC | cloning, gDNA→pBG103 |
| YcaQ_RP_SalI | TATACCGTCGACTTATGCGACGGGGTCTAT | cloning, gDNA→pBG103 |
| YcaQ_FP_NcoI | TGATGACCATGGATGTCGCTGCCGCACCTC | cloning, gDNA→pSF-OBX11 |
| YcaQ_RP_XbaI | TGATGATCTAGATTATGCGACGGGGTCTAT | cloning, gDNA→pSF-OBX11 |
| EndoIV_FP_BamHI | TGATGAGGATCCATGAAATACATTGGAGCG | cloning, gDNA→pHD116 |
| EndoIV_RP_SalI | TGATGAGTCGACTCAGGCTACCGCTTTTTC | cloning, gDNA→pHD116 |
| Ada_FP | AACGACCCAGCTCACAAT | qPCR primer |
| Ada_RP | GCATGACGGCTAAACAATTCC | qPCR primer |
| AlkA_FP | CCCGGTATTGTCATTGGTAAGG | qPCR primer |
| AlkA_RP | CAGACCCGCAGGCATTAAA | qPCR primer |
| GapA_FP | CGGTACCGTTGAAGTGAAAGA | qPCR primer |
| GapA_RP | ACTTCGTCCCATTTCAGGTTAG | qPCR primer |
| LexA_FP | CTGTTGCAGGAAGAGGAAGAA | qPCR primer |
| LexA_RP | GGAAGGATCGACCTGATAATGAC | qPCR primer |
| UvrA_FP | CGCACGGACGATTACTGATAAA | qPCR primer |
| UvrA_RP | CGAAGGCGTGCATAATA | qPCR primer |
| Tag_FP | GAGTCAGGACCCGCTTTATATT | qPCR primer |
| Tag_RP | GGACGGTGATCCACGATAAT | qPCR primer |
| YcaQ_FP | AGGTGATGGTGATTGAACGG | qPCR primer |
| YcaQ_RP | ATGATTTCTGCTTCTGTTTGCG | qPCR primer |
| 7mG_Top | FAM-CACCACTACACC(7mG)ATTCCTTACAAC | base excision, Fig. 1, 5C |
| 7mG_Bottom | GTTGTAAGGAATCGGTGTAGTGGTG | base excision, Fig. 1, 5C |
| AZB_Top | FAM-AAAAATAAAAGCCAAATAAAAATAAA | base excision, Fig. 2, 5A |
| AZB_Bottom | Cy5(TTTATTTTTATTTGGCTTTTATTTTTT | base excision, Fig. 2, 5A |
| NM_Top | FAM-AAAAATAAAAGTCAAATAAAAATAAA | base excision, Fig. 3,4, 5A |
| NM-Bottom | Cy5-TTTATTTTTATTTGACTTTTATTTTT | base excision, Fig. 3,4, 5A |
| AP_DNA | GTTGTAAGGAUTCGGTGTAGTGGTC | base excision, Fig. 5C |
| AP_DNA_FAM | FAM-GTTGTAAGGAUTCGGTGTAGTGGTC | base excision, Fig. S4B |

[a] FAM, 6-carboxyfluorescein; Cy5, cyanine 5

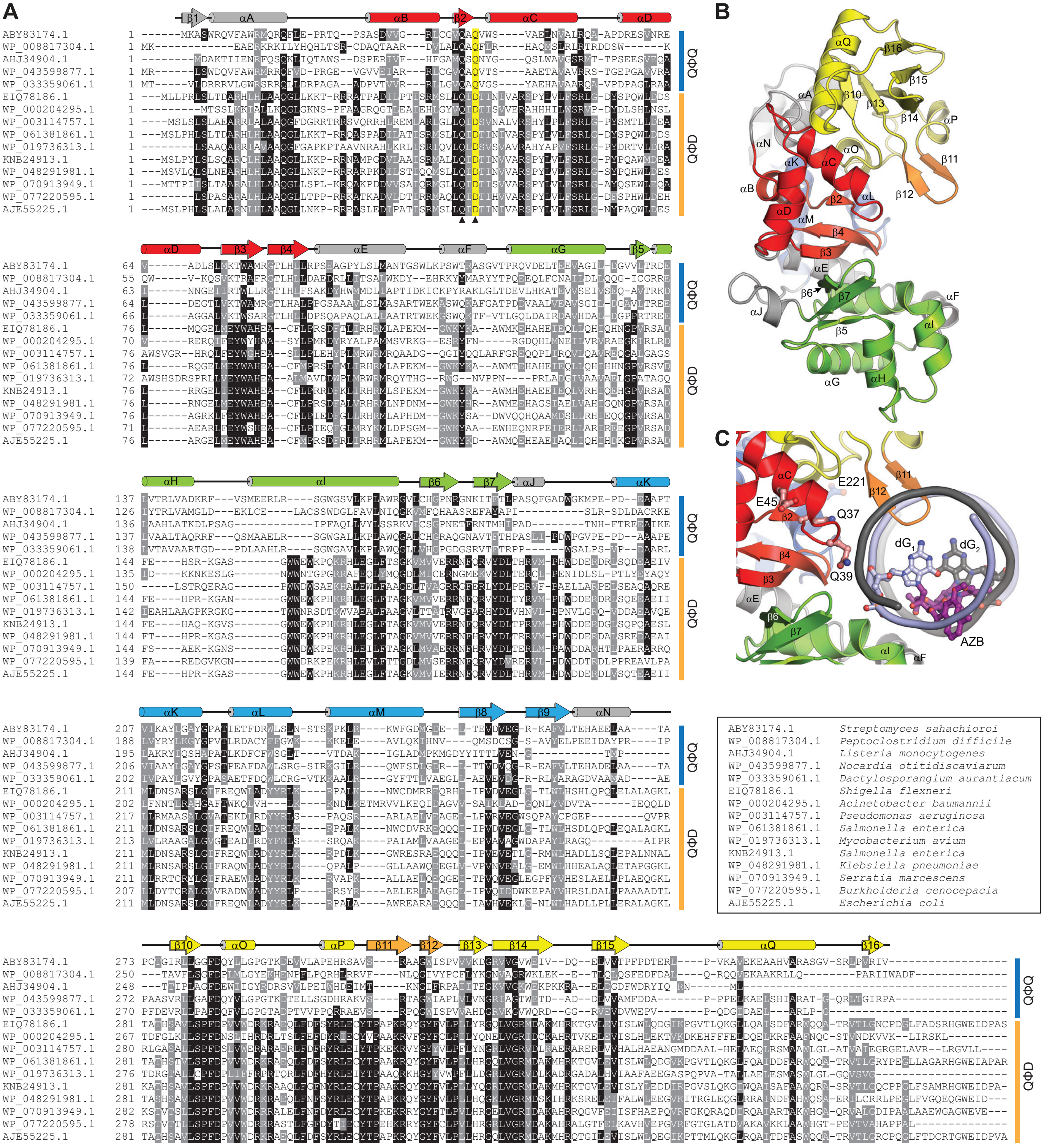


**Fig. S1. Homology of AlkZ orthologs.** (A) Sequence alignment between representative QΦQ and QΦD proteins in the HTH_42 superfamily. The catalytic residues in the QΦQ and QΦD motifs are highlighted with triangles below the sequences. (B) Crystal structure of Streptomyces AlkZ (Mullins et al, 2017, PNAS 114: 4400-4405). (C) Hypothetical model of AlkZ bound to AZB-ICL-DNA.

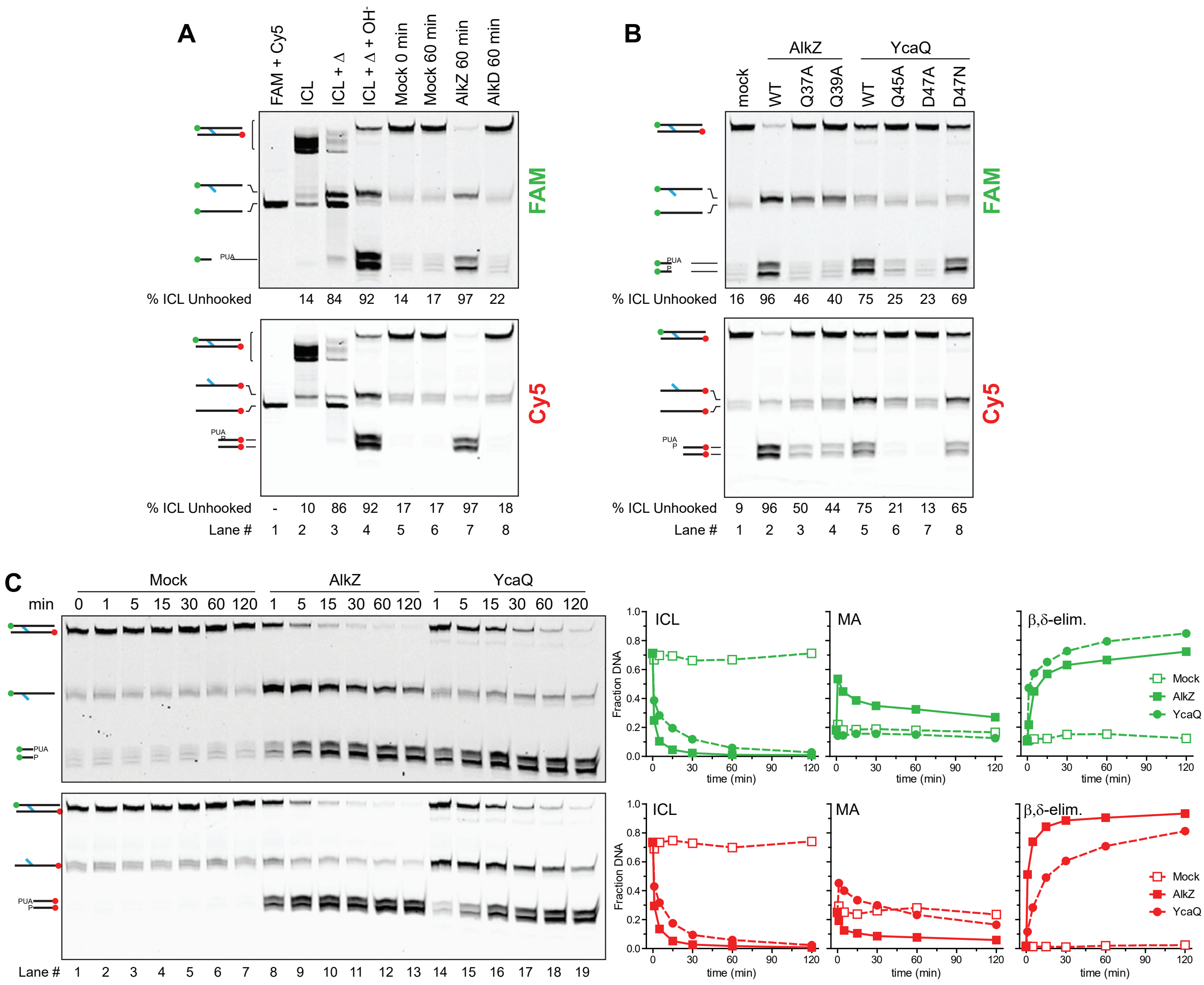


**Fig. S2. Raw data from azinomycin B ICL unhooking assays.** (A) Individual FAM and Cy5 channels for the gel shown in Fig. 2D. Denaturing PAGE of AZB-ICL-DNA substrate treated with either heat (Δ), heat and hydroxide (Δ +OH$^-$), reaction buffer (mock), or AlkZ or AlkD enzymes for the specified amount of time. The gel was imaged for FAM (left) or Cy5 (right) fluorescence. The double-stranded AZB-ICL substrate runs at the top of the gel, intact single strands (containing either monoadducts or not) liberated by ICL unhooking run half-way down the gel, and the β- and β,δ-elimination products arising from alkaline hydrolysis of abasic sites run toward the bottom. ICLs shown in lanes 2 and 3 migrate faster in the gel than those in lanes 4-8 because they were not treated with NaOH in the workup and thus retain positive charge. PUA, 3′-phosphor-α,β-unsaturated aldehyde (β-elimination product), P, 3′-phosphate (β,δ-elimination product). The percent β- and β,δ-elimination products are reported below the gel. (B) Individual FAM and Cy5 channels for the gel shown in Fig. 2E (C) Individual FAM and Cy5 channels for the gel shown in Fig. 2F. The plots to the right quantify the intensities of gel bands corresponding to ICL, monoadduct (MA), and β/δ-elimination products.

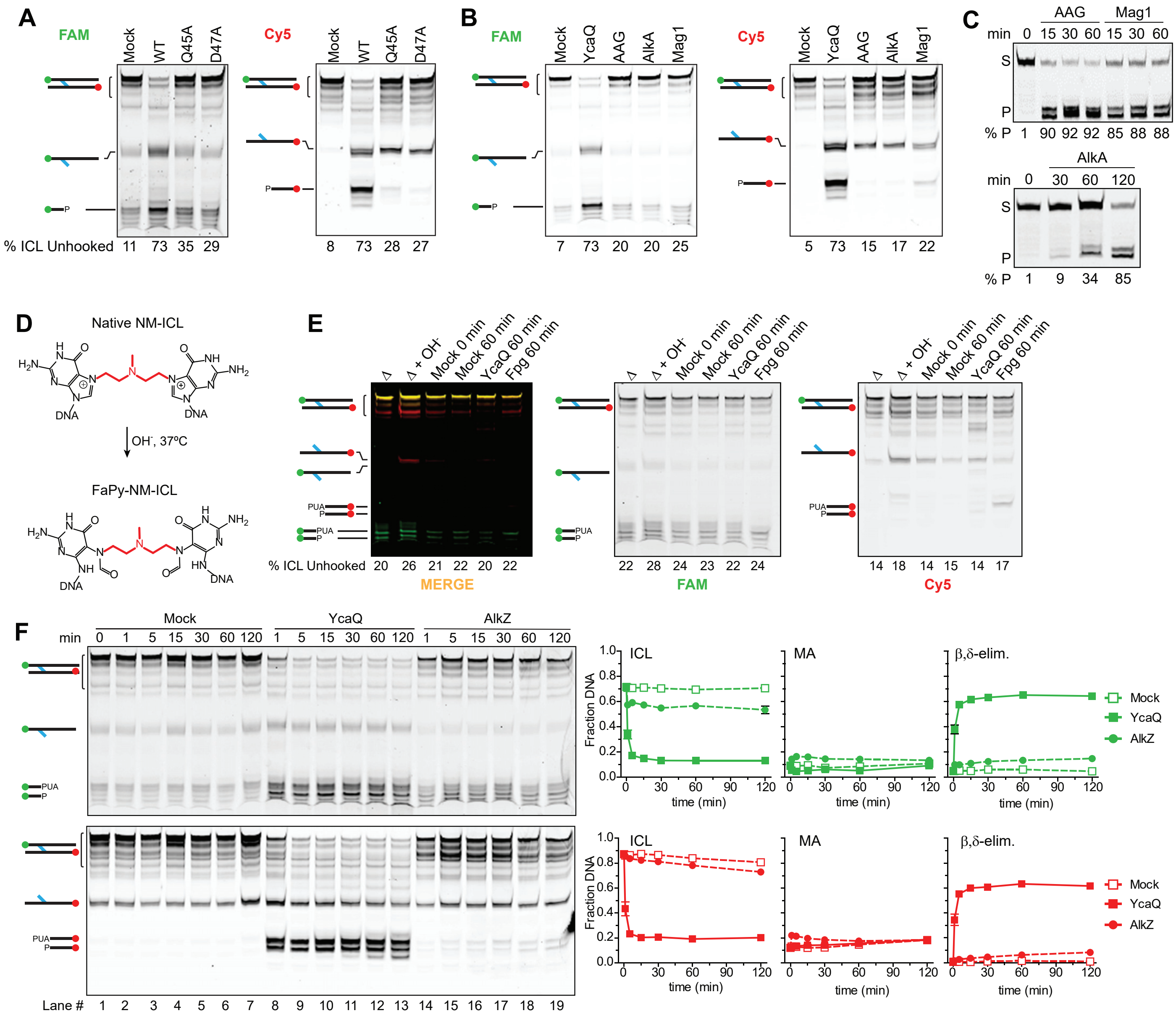


**Fig. S3. Raw data from 5-atom NM-ICL excision assays.** (A) Individual FAM and Cy5 channels for the 5-atom NM-ICL experiment shown in Fig. 3B. Denaturing PAGE of 5-atom-NM-ICL-DNA substrate treated with either no protein (mock) or WT or mutant YcaQ, followed by EndoIV cleavage of AP sites. Positions of bands corresponding to monoadduct and EndoIV cleavage products on the gel are indicated. The percent of ICL unhooked is quantified below. (B) Individual FAM and Cy5 channels for the gel shown in Fig. 3C. NM-ICL-DNA substrate was treated with either no protein (mock), YcaQ, Homo sapiens AAGΔ83, *Escherichia coli* AlkA, or *Schizosaccharomyces pombe* Mag1, followed by EndoIV cleavage. The percent of ICL unhooked is quantified below. (C) Denaturing PAGE separation of d7mG-DNA substrate (S) and nicked abasic-DNA product (P) after incubation of substrate with either AAGΔ83, Mag1, or AlkA for the specified time, followed by alkaline hydrolysis. The percent of β,δ-elimination (product) is quantified below. (D) Hydroxide-catalyzed conversion of cationic NM-ICLs to neutral, ring-opened-FaPy-NM-ICLs. (E) Individual FAM and Cy5 channels and their overlay of a denaturing PAGE containing FaPy-NM-ICL-DNA substrate, monoadducts, and nicked abasic-DNA products after treatment with buffer (mock), YcaQ, or E. coli Fpg for the specified time, followed by alkaline hydrolysis. Heat-mediated depurination (lanes 1-2) serve as a positive control for FaPy adduct generation. The percent of ICL unhooked is quantified below. (F) Individual FAM and Cy5 channels for the gel shown in Fig. 3D. Denaturing PAGE of FaPy-NM-ICL-DNA substrate after treatment with buffer (mock), YcaQ, or AlkZ for the specified time, followed by alkaline hydrolysis. ICL substrate, unhooked monoadduct (MA) strands, and nicked abasic-DNA β,δ-elimination products are quantified to the right. Error bars represent SEM (n=3).

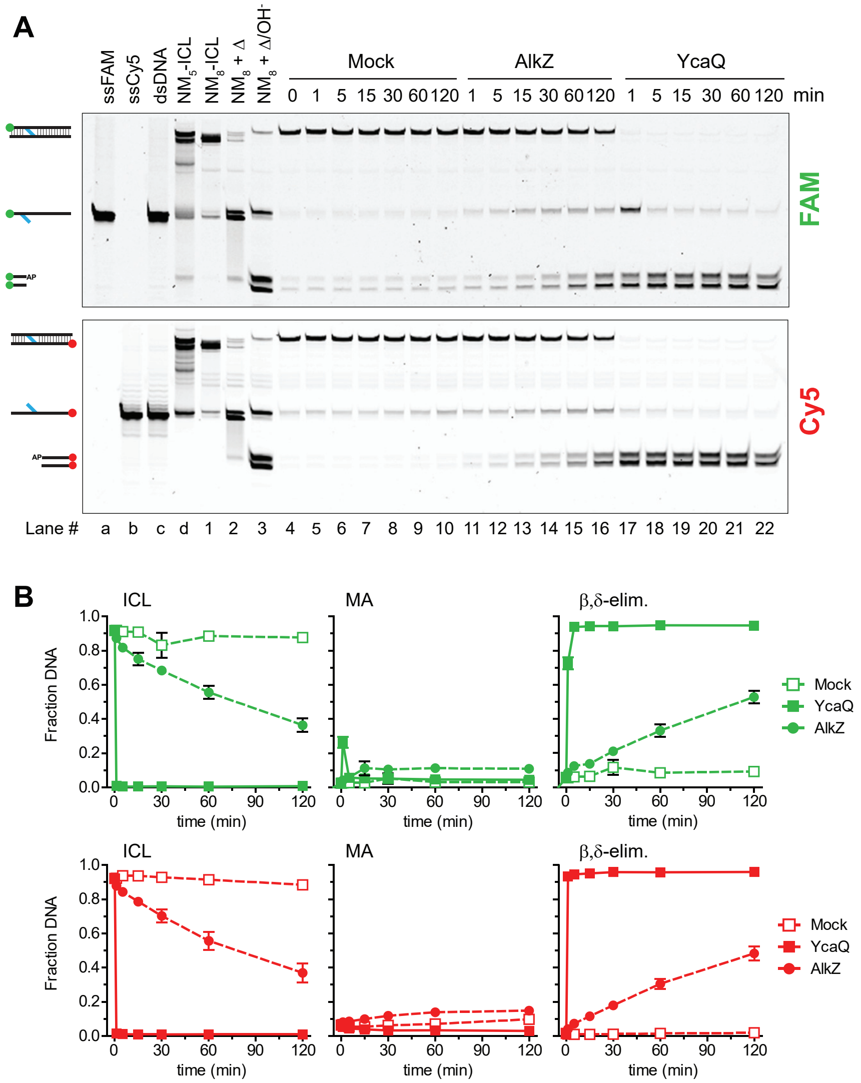


**Fig. S4. Raw data from 8-Atom NM-ICL excision assays.** (A) Individual FAM and Cy5 channels for the 8-atom NM-ICL-DNA experiment shown in Fig. 4B. Denaturing PAGE of the 8-atom NM-ICL-DNA substrate after treatment with buffer (mock), YcaQ, or AlkZ for the specified time, followed by alkaline hydrolysis. Single-stranded (ss), double-stranded (ds), and crosslinked standards are shown in lanes 1-7. (B) Quantification of ICL substrate, unhooked monoadduct (MA) strands, and nicked abasic-DNA β,δ-elimination products from the gels shown in panel A. Error bars represent SEM (n=3).

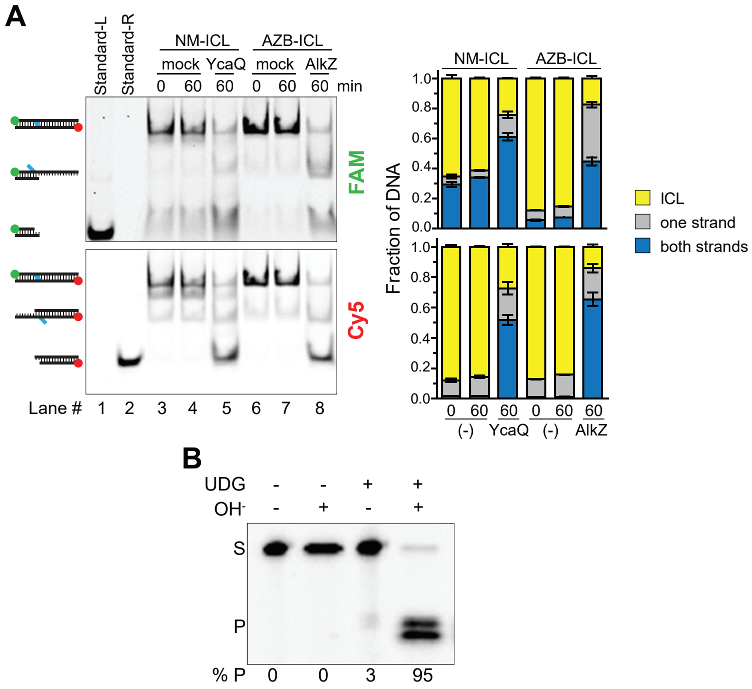


**Fig. S5. Individual Filters for Native PAGE Gel of ICL Unhooking and AP Site Generation.** (A) Individual FAM and Cy5 channels for the gel shown in Fig. 5A. Quantification of the total fluorescent signal for the fraction of ICL, single- and double-nicked EndoIV cleavage products from the gels are plotted on the right. (B) Denaturing PAGE of dU-DNA substrate (S) and nicked AP-DNA products (P) after treatment with or without UDG (+/-) and/or hydroxide (+/-). The percent of β,δ-elimination product is quantified below.

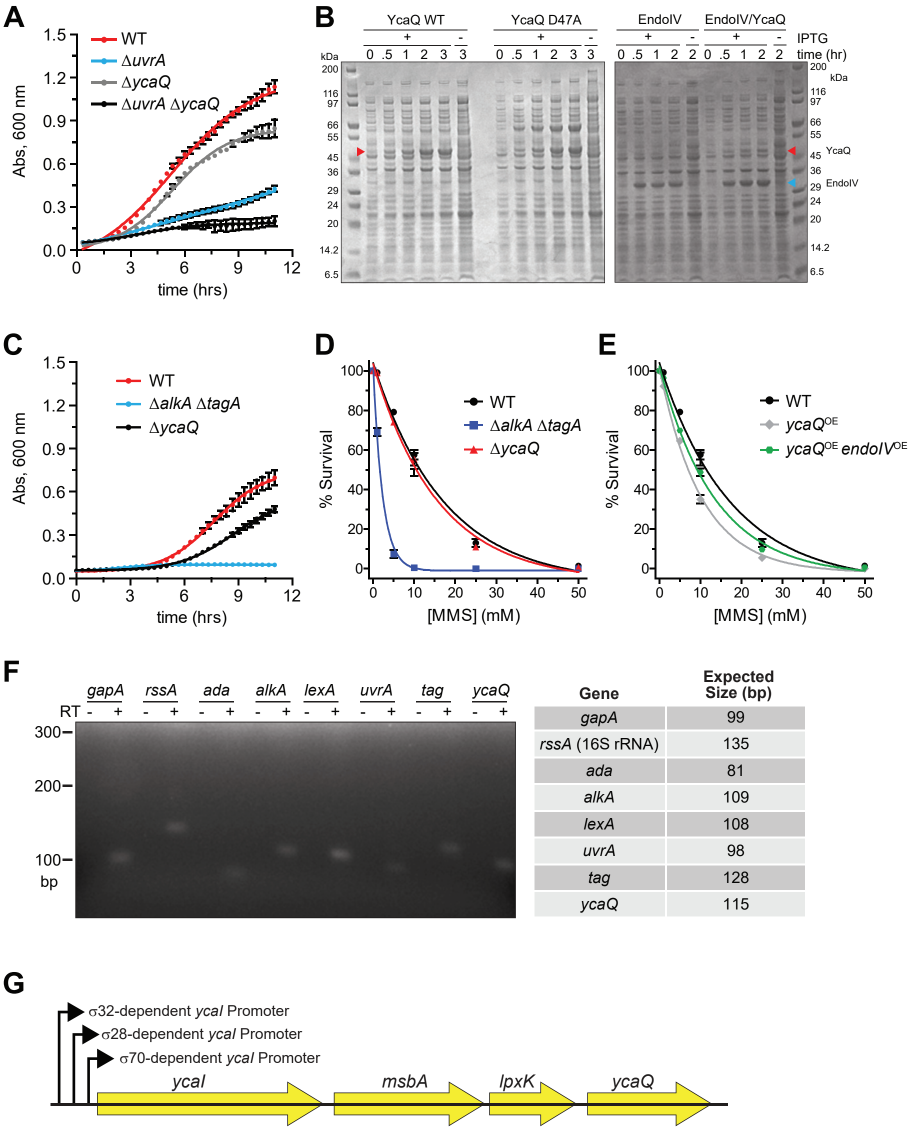


**Fig. S6. Growth curves and drug sensitivity of ycaQ mutant cells.** (A) Growth curves of wild type or mutant *E. coli* strains in LB media containing 33 μM mechlorethamine. Values are mean ± SEM (n=3). (B) SDS PAGE of YcaQ WT and D47A and EndoIV and YcaQ/EndoIV overexpression. Red and blue triangles denote YcaQ (47.7 kDa) and EndoIV (31.5 kDa), respectively. (C) Growth curves of *E. coli* strains in LB media containing 5 mM MMS. (D,E) Colony dilution sensitivity of *E. coli* deletion strains (D) or wild-type *E. coli* overexpressing ycaQ or both ycaQ + endoIV (E) exposed to increasing concentrations of MMS. Values are mean ± SEM (n=3). Percent survival is in relation to untreated WT K-12 cells. (F) 3% agarose gel of RT-PCR primer extension products after 25 thermal cycles. (+) reactions included reverse transcriptase; (-) reactions excluded RT. Positions of the 100-base pair marker are indicated to the left of the gel. The expected sizes of the extension products are shown in the table to the right. (G) Predicted operon structure for YcaQ from the EcoCyc database data. Putative δ-dependent promoters are labeled in front of the operon and are predicted based on consensus sequence similarities to known promoters.
